# Responsibility for managing musculoskeletal disorders – A cross-sectional postal survey of attitudes

**DOI:** 10.1186/1471-2474-9-110

**Published:** 2008-08-05

**Authors:** Maria EH Larsson, Lena A Nordholm

**Affiliations:** 1 Department of Clinical Neuroscience and Rehabilitation/Physiotherapy, Institute of Neuroscience and Physiology, The Sahlgrenska academy at University of Gothenburg, Box 455, SE 405 30 Gothenburg, Sweden; 2 The Vårdal Institute, Lund University, P.O. Box 187, SE-221 00 Lund, Sweden; 3 Research and Development unit, Primary care, County of South Bohuslän, Box 2004, SE 431 02 Mölndal, Sweden; 4 University College of Borås, SE 501 90 Borås, Sweden

## Abstract

**Background:**

Musculoskeletal disorders are a major burden on individuals, health systems and social care systems and rehabilitation efforts in these disorders are considerable. Self-care is often considered a cost effective treatment alternative owing to limited health care resources. But what are the expectations and attitudes in this question in the general population? The purpose of this study was to describe general attitudes to responsibility for the management of musculoskeletal disorders and to explore associations between attitudes and background variables.

**Methods:**

A cross-sectional, postal questionnaire survey was carried out with a random sample of a general adult Swedish population of 1770 persons. Sixty-one percent (n = 1082) responded to the questionnaire and was included for the description of general attitudes towards responsibility for the management of musculoskeletal disorders. For the further analyses of associations to background variables 683–693 individuals could be included. Attitudes were measured by the "Attitudes regarding Responsibility for Musculoskeletal disorders" (ARM) instrument, where responsibility is attributed on four dimensions; to myself, as being out of my hands, to employers or to (medical) professionals. Multiple logistic regression was used to explore associations between attitudes to musculoskeletal disorders and the background variables age, sex, education, physical activity, presence of musculoskeletal disorders, sick leave and whether the person had visited a care provider.

**Results:**

A majority of participants had internal views, i.e. showed an attitude of taking personal responsibility for musculoskeletal disorders, and did not place responsibility for the management out of their own hands or to employers. However, attributing shared responsibility between self and medical professionals was also found.

The main associations found between attitude towards responsibility for musculoskeletal disorders and investigated background variables were that physical inactivity (OR 2.92–9.20), musculoskeletal disorder related sick leave (OR 2.31–3.07) and no education beyond the compulsory level (OR 3.12–4.76) increased the odds of attributing responsibility externally, i.e placing responsibility on someone or something else.

**Conclusion:**

Respondents in this study mainly saw themselves as responsible for managing musculoskeletal disorders. The associated background variables refined this finding and one conclusion is that, to optimise outcome when planning the prevention, treatment and management of these disorders, people's attitudes should be taken into account.

## Background

Musculoskeletal conditions are without doubt a major burden on individuals, health systems and social care systems, where low back pain is the most prevalent musculoskeletal condition [1]. Systematic reviews of musculoskeletal disorders such as low back and neck pain show that this pain is rarely a symptom of serious disease but that most people will be affected by it at some point in life [2]. Since recurrences are to be expected, patients can benefit from a plan for managing flare-ups [3]. As the health care system often provides symptomatic alleviation but rarely cures, handling of musculoskeletal problems needs to be based on a better integration of perspectives, including those of the patient [4]. Results of a randomized trial of a cognitive-behavioural program for enhancing back pain self-care point to the potential for patients to assume greater responsibility for managing back pain than is often expected by health care professionals [5]. This was also discussed by Jamison in an Australian case study of perceptions of responsibility for "getting well" which showed congruence within patient-chiropractor dyads in only 29%. The discrepancy in the perceptions of responsibility was largely attributable to patients believing they should take greater responsibility than was expected by their chiropractors. Practitioners who underestimate the willingness of patients to take substantial personal responsibility for their health may overlook an opportunity to promote health [6]. A British study showed that the problem of managing back pain might be reduced by closing the gap between the public's expectations and what is recommended in guidelines [7]. According to Harter (1995), the most important objective of therapeutic and health promotion measures should be to teach patients to assume responsibility for their own health [8]. As musculoskeletal disorders affect so many people at some point in life, it would be of interest and importance to study a general population's attitudes in the matter of responsibility for musculoskeletal disorders. Are people prepared to assume responsibility for prevention, treatment and management of these disorders, or do they feel that the management of musculoskeletal disorders is chiefly the responsibility of medical professionals or employers or that there is nothing they themselves can do? Could information of the general population's attitudes be helpful when discussion where and for whom it would be most effective to work with preventative or promotion activities? Can associations be found with background variables that could be of interest when planning preventive care as well as treatment of musculoskeletal disorders?

We believe that, for better management of musculoskeletal disorders and possibilities of increased self-care interventions, it is imperative to be acquainted with attitudes towards the responsibility for musculoskeletal disorders. People have different ways of ascribing responsibility and causality (locus of control) in their lives. Those with an internal locus of control see themselves responsible for the outcomes of their own actions. Someone with an external locus of control sees environmental causes and situational factors as being more important than internal ones. This concept was originally developed by Julian Rotter in the 1960s [9] but has been used widely in health-specific instruments such as the Multidimensional Health Locus of Control Scales (MHLC) [10].

Attitudes are thought to influence feelings and behaviour [11]. Many studies with demonstrated effectiveness in the treatment of musculoskeletal disorders or disability, often include a cognitive-behavioural component aiming at increasing self-efficacy [12,13], and according to the theory of planned behaviour [14], attitude regarding the behaviour is one of the determinations of intention, which in turn can predict behaviour in relation to the object of concern.

Larsson and Nordholm developed an instrument called "Attitudes regarding responsibility for musculoskeletal disorders" (ARM) [15]. Although the ARM instrument was inspired from the MHLC [10], the ARM instrument specifically measures attitudes towards responsibility for musculoskeletal disorders and not control of general health as the MHLC. With the ARM instrument, on four dimensions, people attribute responsibility internally, as a self-active process, or externally as "out of their hands", to be a matter for employers or (medical) professionals. If people's attitudes were better known, health planners and care providers could obtain a better opportunity to plan for preventive actions or treatment and make more efficient plans for managing these problems. Attitudes towards responsibility for managing musculoskeletal disorders have not yet been investigated in a general population.

The aim of this study was to describe a population's general attitudes towards responsibility for musculoskeletal disorders. A further aim was to explore the associations between attitudes regarding responsibility for musculoskeletal disorders and the background variables age, sex, education, physical activity, presence of musculoskeletal disorders, sick leave and visits to care providers.

This article reports information on who people feel bear the greatest responsibility for prevention, treatment and management of musculoskeletal disorders and which of the studied background variables are associated with increased odds of placing responsibility on someone or something else but the person him or herself.

## Method

The study was carried out as a cross-sectional postal survey. The SPSS statistical program (Statistical Package for the Social Sciences, Chicago IL) version 13.0 for Microsoft Windows was used to extract a random sample of one percent (1770 persons) of the adult population (18 years or older) from the population register of each of the eight municipalities belonging to the Primary Care district of south Bohuslän, in the vicinity of the city of Gothenburg (Sweden).

Participants were mailed written information, a questionnaire and a stamped self-addressed envelope. Part one of the questionnaire contained the "Attitudes regarding Responsibility for Musculoskeletal disorders" (ARM) instrument [15]. Part two included questions on background variables; *age, sex *and *education *categorised as university, high school, compulsory school or "other" level that included adult education programs and vocational training. *Physical activity *was assessed on a four-graded scale from none/very little to at least three times a week. *Musculoskeletal disorders *during the last three months were stated using check boxes for nine locations of the body. *Sick leave *implied more than seven days during the most recent 12 months that required a doctor's certificate (yes/no format) with additional check boxes for the reason of sick leave and *visits to care providers *were reported for the last three months also using check boxes providing six different care providers.

The questionnaires were uncoded, and thus answered anonymously, and one reminder including the full questionnaire was sent to all the participants after seven weeks. Respondents consented to participate by returning the completed questionnaire. The study was approved by the Ethics Committee of University of Gothenburg.

The ARM instrument consists of 15 items on four dimensions, six items attribute responsibility to myself; the dimension is called "responsibility self-active", three items attribute "responsibility to be out of my hands", three items attribute "responsibility to employers" and three items attribute "responsibility to (medical) professionals" (see also Additional file 1). Each item is rated on a six-point Likert-type scale from 1 (strongly disagree) to 6 (strongly agree). Internal attitude regarding responsibility for musculoskeletal disorders implies that the individual takes an active part in the prevention, treatment or management of musculoskeletal disorders. External attitude implies that individuals turn over responsibility to someone or something without regarding themselves as active in the prevention, treatment or management of musculoskeletal disorders [15]. In calculating scores, internal items (the items of the "responsibility self active" dimension) were reversed, thus expressing degrees of externality by increasing scores (possible range of "responsibility self-active" 6–36, of "responsibility out of my hands", "responsibility employer" and "responsibility (medical) professionals" 3–18) [15]. Internal consistency (Cronbach's alpha) for ARM have been reported to range from .69 to .85 on the four dimensions, acceptable stability was reported and construct and content validity were supported [15].

Data were analysed using the SPSS. Descriptive statistics were used to describe participants' general attitudes towards responsibility for musculoskeletal disorders in the four dimensions of "responsibility self-active", "responsibility out of my hands", "responsibility employer" and "responsibility (medical) professionals". Multiple logistic regression analyses with stepwise, backward removal of covariates (Wald) on the .10 level were used to analyse the association between attitudes towards musculoskeletal disorders (as measured with ARM) and background variables; *age, sex, education, physical activity, musculoskeletal disorders, sick leave *and *visits to care providers*. Associations were expressed as odds ratios (OR) with 95% confidence intervals (95% CI). Separate analyses were made for each of the four dimensions as the dependent variable. The sample's upper quartile for the dimension was chosen as the cut-off score: "responsibility self-active" ≥ 17 p, "responsibility out of my hands" ≥ 8 p, "responsibility employer" ≥ 9 p and "responsibility (medical) professionals" ≥ 14 p. Thus outcome was determined by the 25% with the most external attitude.

The models were thereafter controlled for interaction effects between the musculoskeletal disorders variable and each of the other background variables in all four dimensions.

Comparisons between two groups for internal missing analyses were made with t-test for numerical data, Mann-Whitney U-test for data on ordinal level and with chi-square test for categorical data on nominal or ordinal level.

## Results

Questionnaires were received from 1082 persons (61%) of the sample. Age ranged from 18 to 99 years old, with a mean of 50 years (sd 16). Table 1 shows a presentation of the background variables in the present sample and comparative statistics of South Bohuslän and Swedish national data.

**Table 1:** Descriptives of the present sample's background variables and comparative statistics of South Bohuslän and Swedish national data (n = 1082).

	Present sample	South Bohuslän	National
Sex			
• Women	51%	50%^1^	50.5%^1^
• Men	49%	50%^1^	49.5%^1^
Age (year)			
• 18–44	38%	46%^1^	45%^1^
• 45–64	43%	35%^1^	33%^1^
• 65+	19%	19%^1^	22%^1^
Education			
• Compulsory	20%	18%^1^	19%^1^
• High school + other	47%	49%^1^	48%^1^
• University	32%	32%^1^	31%^1^
• Missing	1%		
Physical activity			*
• Perform at least 3 times/week	33%		
• Perform 1–2 times/week	31%		
• Perform now and then	24%	*30%^a^*	
• Perform none or very little	10%	*30%^a^*	
• Missing	2%		
Sick-leave			
• Sick-leave total	17%	13–17%, mean 15%^2^	15%^2^
• MSD related sick-leave	7%	* ^3^	* ^3^
• Missing	4%		
Presence of musculoskeletal disorders (MSD)			*
• No musculoskeletal disorders	15%		
• Suffered from musculoskeletal disorders	59%	*47–56% (7 out of 8 municipalities)*^a^	
• Missing	26%		
Visits to care provider			*
• No visits	53%		
• Visited	28%	*39–46%(7 out of 8 municipalities)*^a^	
• Missing	19%		

^1^[Statistics Sweden]

^2 ^[Swedish social insurance agency]

^3 ^[Swedish social insurance agency] Diagnosis was not registered the investigated period.

^a ^[Life and Health 2003, Region Västra Götaland] *Regional survey report, not statistics*

*no comparative data available

Approximately 10% of the respondents answered the reminder; these did not differ significantly on any of the variables included in the questionnaire.

### Generalized attitudes regarding responsibility for musculoskeletal disorders

**Figure 1:**
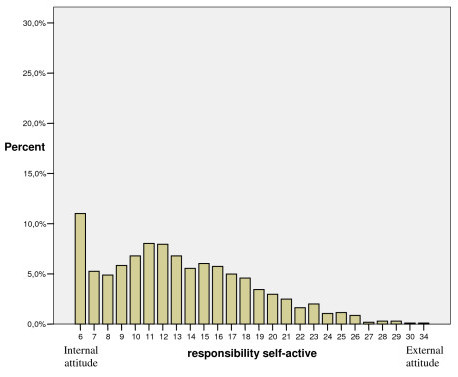
Distribution of participants' scores in the "responsibility self-active" dimension given in percent (n = 1045).

**Figure 2:**
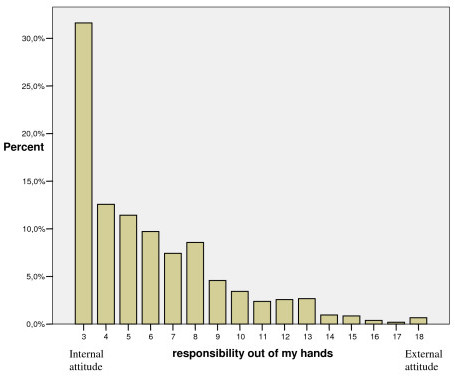
Distribution of participants' scores in the "responsibility out of my hands" dimension given in percent (n = 1050).

**Figure 3:**
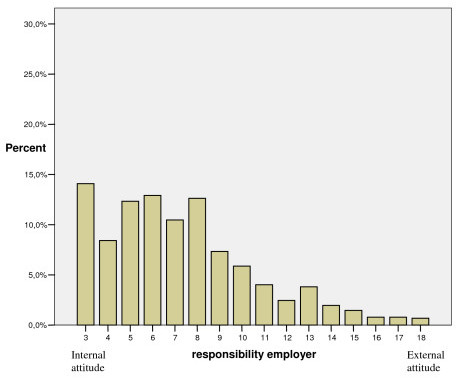
Distribution of participants' scores in the "responsibility employer" dimension given in percent (n = 1022).

As shown in Figures 1, 2, 3, each of the dimensions "responsibility self-active", "responsibility out of my hands" and "responsibility employer" had a positively skewed distribution, implying that a majority of the participants showed an internal view of responsibility for musculoskeletal disorders and did not place responsibility to be out of their hands or on employers to any great extent.

**Figure 4:**
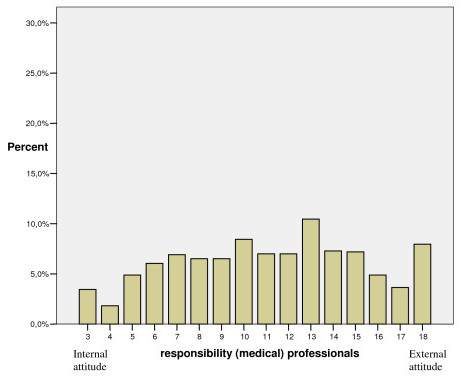
Distribution of participants' scores in the "responsibility (medical) professionals" dimension given in percent (n = 1043).

A more equal distribution was seen in the dimension of "responsibility (medical) professionals", which implied shared responsibility between the individual and medical professionals (Figure 4).

The correlation coefficients between the four dimensions ranged from r_s _.177 to .377 (p < 0.001)

#### External missing

As the respondents were anonymous, no description and comparison of non-respondents (external missing analysis) could be made, although the collected sample was compared to municipal and national data for *sex, age *and *education *[Statistics Sweden], *sick leave *[Swedish social insurance agency], *presence of musculoskeletal disorders *and *physical activity *[Life and Health 2003, Region Västra Götaland] (see Table 1). The collected sample was somewhat over-represented in the "middle-aged" group.

### Associations between attitudes towards responsibility for musculoskeletal disorders and background variables

Multiple logistic regression handles only completed forms. If data were missing in any variable, the individual was excluded. As can be seen in Table 1, the background variables *visited care provider *and *presence of musculoskeletal disorders *had many internal missing data and thereby dramatically reduced the number of individuals leaving approximately 690 cases for analyses of associations.

Table 2 shows the results of the multiple logistic regression of association of background variables with the four dimensions in ARM.

**Table 2:** Multiple logistic regressions of associations of background variables with each of the four dimensions of Attitudes regarding responsibility for musculoskeletal disorders (ARM).

Variable	Dimension							
	"Responsibility Self Active" ≥ 17 p Included in analysis n = 693, above cut-off n = 176		"Responsibility Out of my hands" ≥ 8 p Included in analysis n = 693, above cut-off n = 156		"Responsibility Employer" ≥ 9 p Included in analysis n = 683, above cut-off n = 174		"Responsibility (Medical) Professionals" ≥ 14 p Included in analysis n = 692, above cut-off n = 182	

	OR	CI (95%)	OR	CI (95%)	OR	CI (95%)	OR	CI (95%)
								
**Age **(years old)								
18–40 (ref)	1.00		1.00		1.00		1.00	
41–64	.73	.49; 1.08	.73	.47; 1.14	**.49**	**.32; .74**	1.09	.71; 1.69
>65	**. 43**	**.22; .83**	1.26	.69; 2.28	.80	.44; 1.44	**2.49**	**1.41; 4.40**
								
**Gender**								
Male (ref)					1.00			
Female					**1.49**	**1.03; 2.16**		
								
**Education**								
University (ref)			*1.00*		1.00		1.00	
High school			*1.30*	*.81; 2.11*	1.25	.81; 1.95	**2.15**	**1.34; 3.47**
Compulsory school			***4.10***	***2.35; 7.15***	**3.12**	**1.81; 5.40**	**4.76**	**2.73; 8.29**
Other			*1.93*	*.93; 3.98*	1.94	.98; 3.88	**3.30**	**1.67; 6.55**
								
**Physical activity**								
Perform at least 3 times/week (ref)	1.00		1.00					
Perform 1–2 times/week	**2.66**	**1.58; 4.49**	1.13	.70; 1.82				
Perform now and then	**6.44**	**3.81; 10.89**	1.57	.96; 2.57				
Perform none or very little	**9.20**	**4.58; 18.50**	**2.92**	**1.50; 5.69**				
								
**Presence of musculoskeletal disorders (MSD)**								
No musculoskeletal disorders (ref)	1.00				1.00		1.00	
Suffered from musculoskeletal disorders	**2.78**	**1.58; 4.89**			.66	.43; 1.01	**.42**	**.27; .65**
								
**Sick-leave**								
No sick-leave (ref)	1.00		1.00		1.00			
Sick-leave but not for MSD	1.44	.78; 2.65	1.27	.66; 2.43	1.78	.99; 3.22		
MSD related sick-leave	**2.55**	**1.18; 5.48**	**2.31**	**1.08; 4.91**	**3.07**	**1.48; 6.39**		
								
**Visits to care provider**								
No visits (ref)							1.00	
Visited							**2.07**	**1.40; 3.05**

Italics: Interaction effect with MSD

The dependant value was scoring in the upper quartile of the population, thus belonging to the group of people (25%) with the most external attitude. Significant associations are in bold type.

Having musculoskeletal disorders, being physically inactive and musculoskeletal disorder related sick leave were all strongly associated with the most external attitude in the "responsibility self-active" dimension, implying that individuals did not consider themselves to have an active role in the prevention and management of musculoskeletal disorders. External attitude associated with being physically inactive and musculoskeletal disorder related sick leave were also reflected by the "responsibility to be out of my hands" dimension, compulsory schooling was also associated to the most external attitude in this dimension.

Being female, having a compulsory school education and musculoskeletal disorder related sick leave were associated with placing responsibility on the employer. Being middle-aged, on the other hand, had a negative association with placing responsibility on the employer.

Those who had reached retirement age, people who stated that they visited a care provider and having less than university education at least doubled the odds of placing responsibility externally on medical professionals. Presence of musculoskeletal disorders and on the other hand, decreased the odds of being amongst those with the most external attitudes.

One significant interaction effect with musculoskeletal disorder was found. In the "responsibility out of my hands" dimension we found that a lower level of education showed a strong positive association with externality among those with musculoskeletal disorders in contrast to those without musculoskeletal disorders. A stratified analysis showed that in the group with musculoskeletal disorders (n = 552) OR's for being amongst those with the most external attitude equalled to 5.57, 1.38 and 2.46 for compulsory, high school and other education compared to university education (p < .001, .27, .03). The corresponding OR's for those without musculoskeletal disorders (n = 141) are given by .76, .84, .55 (p > 0.5).

Medians and quartiles for the background variables *presence of musculoskeletal disorders *and *sick leave *are provided in Table 3.

**Table 3:** Medians (Md) and quartiles (Q1;Q3) for the background variables *Presence of musculoskeletal disorders *and *Sick-leave *for those included in the regression analyses.

	"Responsibility Self Active"		"Responsibility Out of my hands"		"Responsibility Employer"		"Responsibility Medical Professionals"	
	Md	Q1; Q3	Md	Q1; Q3	Md	Q1; Q3	Md	Q1; Q3
**Presence of musculoskeletal disorders (MSD)**								
- No musculoskeletal disorders	11	7; 14	5	3; 7.5	7	5; 9	13	9; 16
- Suffered from musculoskeletal disorders	13	9.25; 17	5	3; 7	6	5; 8	10	7; 13
								
**Sick-leave**								
- No sick-leave	12	9; 16	5	3; 7	6	4; 8	11	7, 14
- Sick-leave but not for MSD	14.5	10; 18	5	3; 7	7	5; 9	11	8, 13
- MSD related sick-leave	15	8; 19	7	3; 10	8	6; 11	11	7.5; 15

#### Internal missing analyses

The two background factors *presence of musculoskeletal disorders *and *visited care provider *were responsible for 28% out of the 36% respondents that were missing in the analyses of associations. If these two variables were excluded from the multiple logistic regressions, giving rise to a larger sample (n = 994), the remaining variables still showed similar associations with the outcome.

There were no statistically significant differences in overall presence of musculoskeletal disorders and visited care provider between those included in the regression analyses, (complete respondents, n = 693) and those not included in the analyses, partial respondents, (presence of musculoskeletal disorders, n = 96, p = .37; visited care provider n = 173, p = .72)

More women were among the partial (missing) respondents (p = .003). The partial respondents were somewhat older (p < .001), were lower educated (p < .001), were less physical active (p = .01), and more likely to been on sick leave (p < .001). They also had more external attitudes wrt. "responsibility out of my hands", "responsibility employer" and "responsibility medical professionals" (p < .001).

## Discussion

### Generalized attitudes regarding responsibility for musculoskeletal disorders

The issue of responsibility for an individual's health or illness has no definite answers but many viewpoints. Many patients and most physicians behave as though doctors have the primary responsibility. Others strongly believe that the ultimate responsibility for health lies or should lie firmly with the individual. Still others believe that no one is ultimately responsible for health or illness [16].

A majority of the participants in the present study had internal views regarding responsibility for musculoskeletal disorders, i.e. they thought that they themselves should take responsibility and not place responsibility in the hands of employers or consider the matter to be out of their hands. As the dimensions provide information on separate but closely related constructs an overlap could be expected. An individual who shows internal view regarding out of my hands probably also does so in the other externally directed dimensions as well, but as the dimensions were not highly correlated we found it valuable to report results for the dimensions separately.

That the investigated sample showed internal views could be seen as a positive result, encouraging for public musculoskeletal health interventions. The findings are also consistent with those of Jamison (2000) [6]. That many people expressed the attitude of shared responsibility between themselves and medical professionals can also be considered positive, as individuals who express the belief that their health is controllable are possibly the most adaptive. This belief could be particularly beneficial to those who must cope with a chronic illness [16].

The investigated population probably would benefit from the treatments currently available for musculoskeletal disorders, as these usually include medical professionals involvement but also some degree of self-responsibility [17-19]. Support has been found for the definition of compliance as an active, responsible process in which the patient works to maintain health in close collaboration with health care personnel [20]. Internal scores in the "responsibility self-active" dimension and intermediate scores in the "responsibility (medical) professionals" dimension in the present study show that chances are good for such a process.

However, previous studies have shown that patients with chronic musculoskeletal pain with high agency orientation benefited more from a group learning program with regard to pain reduction and improved pain coping than did those patients with low agency orientation [21]. Studies has also shown that people with weak beliefs in the controllability of their back problem were more likely to have poor clinical outcomes six months after they consulted their doctor [22]. So, there still could be a need of identifying and better targeting psychosocial interventions at those who are at high risk of persistent pain, and are likely to respond better to interventions with a more cognitive-behavioural focus [23].

Also in other fields relationships of attitudes and response to treatment have been found. Galgut and co-workers showed that subjects who perceived their susceptibility to disease as being influenced by powerful external factors or who believed that susceptibility could be controlled by their own actions responded more positively to a plaque control regime than those who considered susceptibility to disease an event of chance [24].

### Associations between attitudes towards responsibility for musculoskeletal disorders and background variables

The choice of using logistic regression was based on the fact that data were mainly on categorical level and the original scaling included too many levels for ordinal regression. A clinical interest in those with most external attitudes and as studies in other fields show that external attitude have associations to poorer health led to the decision of using the upper quartile as cut-off.

The main associations found were that physical inactivity, musculoskeletal disorder related sick leave and no education beyond compulsory level increased the odds of placing responsibility for musculoskeletal disorders externally.

That those with lower socio-economic status tend to have higher external scores, while people with higher socio-economic status and/or beneficial health behaviour, such as regular exercise, tend to have higher internal scores agrees with closely related research areas, such as health locus of control and coping [25-28]. Since physical activity has been shown to be associated to low prevalence of musculoskeletal disorders [29], one might expect people to be physically active to prevent disorders. However, the association between external attitude and physical inactivity as found in the present study suggests that ARM is a mediating variable. In other words, people who do not think they can influence their musculoskeletal disorders (external attitudes) might not bother to exercise.

An interesting finding in the present study was that people who had musculoskeletal disorder related sick leave had two to three times higher odds of scores in the most external group. This may to some degree be consistent with the work of Haldorsen et al. [30] who found low scores on Internal Health Locus of Control Scale to be a dominant variable for those who did not return to work in a 12-month follow-up study. Millet and Sandberg [31] also found that unemployed individuals with an internal orientation (locus of control) had much shorter periods of sick leave than individuals with an external orientation. What could be the rationale for this? Does sick leave lead to a more external approach to musculoskeletal disorders, or is there a higher probability to be on sick leave because of an external attitude? Do the disorder and its consequences force the individual towards externality? The present study can not answer these questions because no cause and effect relationships were studied. They are however of interest for future studies.

Association with gender was found only in the "responsibility employer" dimension. As the ARM instrument is quite new, it remains to be seen whether this result can be replicated. The results agree with those in the multidimensional health locus of control among New Zealand adolescents [32], a Japanese cohort [27] and in people at risk for coronary heart disease in Scotland [33], but are contrary to patients with chronic fatigue syndrome, where gender related differences were not found [34].

In the present study, elderly people most frequently attributed responsibility for musculoskeletal disorders to medical professionals. On the other hand, there was a negative association to scoring externally in the "responsibility self-active" dimension. This could be interpreted such that people place responsibility both on the medical professionals and on themselves. Healthy elderly people have been characterized by an internal health locus of control and high general self-efficacy, which somewhat supports our results [35]. The combination of an external view of medical professionals' responsibility and internal view of self-active responsibility for musculoskeletal disorders might be the most responsive to health advice and education, similar to "believers in control" [16].

The present study has provided some insight in where a general population place responsibility for musculoskeletal disorders. Previous studies have shown that attitudes and beliefs about how to manage musculoskeletal disorders, as for example low back pain, differ from stated official guidelines [7,36] but there has been limited information about people's attitudes regarding the *responsibility *of management of musculoskeletal disorders. Attitudes and behaviour in the matter of management [37,38] are not easily changed but maybe associated background variables found in the present study can be helpful for more directed interventions in the population.

Although the number of respondents (61%) was not completely satisfying, the sample could be seen as fairly socio-demographically representative according to official municipal and national statistics. However, the category "middle-aged" was slightly over-represented. In this group musculoskeletal disorders are quite common, which may have led to a stronger interest in responding.

Even though the questionnaire had a simple yes/no format in questions about presence of musculoskeletal disorders and visits to care-providers, many of the respondents failed to answer these questions resulting in a large number of internal missing cases. However, comparisons between partial and full respondents showed significant differences in some of the background variables but since female sex, low education, inactivity and sick leave have been found to be associated with higher externality in the full analysis, we conclude that the partial respondents show similar associations (even though they can be shown on group level only).

How then, with knowledge of people's attitudes, can we best avoid or decrease the suffering and burden of musculoskeletal disorders? Payton and associates [39] found that the general public needed much more information about what to expect of physical therapy. Patients need an individualised analysis of how they view their role in health care and instruction on how to assume greater responsibility for their care [39]. Von Korff et al. (1997) presented a model where patients and care providers share goals, a sustained working relationship, mutual understanding of roles and responsibilities and requisite skills for carrying out these roles [40]. A randomised trial of a cognitive-behavioural program for enhancing back pain self-care in a primary care setting showed that the self-care intervention led to significantly greater reductions in back-related worry and fear-avoidance beliefs than controls [5]. Further research is needed to study timing and inclusion criteria for interventions that enhance self-care and affect patient outcomes.

Furthermore, we believe that information on perceptions of responsibility for musculoskeletal disorders could help in the development of personalized action plans to manage pain and to make them more specific in preventive care. Where attitudes differ between care providers and the general population, the options are to either go along with common attitudes or challenge them.

Horneij (2001) and co-workers explained their non significantly different results between two interventions such that, as the aetiology of musculoskeletal disorders is multifactorial, a combination of the two programs might be preferable [41]. Perhaps a sub-categorisation, for example on the grounds of attitude, also could have improved the outcome. Haldorsen (2002) and co-workers questioned whether there is a right treatment for a particular patient group when they evaluated comparisons of ordinary treatment, light multidisciplinary treatment and extensive multidisciplinary treatment for long-term sick listed employees with musculoskeletal pain. Their conclusion was that multidisciplinary treatment was effective when given to those most likely to benefit from that treatment and that a simple screening instrument could be a useful clinical tool for allocating patients to the appropriate level of treatment [42]. Another study showed that patients allocated to the intervention that they had expressed a preference for, had clinically important reductions in pain and disability [43]. Smeets and co-workers found significant differences when they compared patients on waiting list with patients who received treatment (either cognitive-behavioural or physical rehabilitation or both) for chronic low back pain. However, no clinically relevant differences between the treatment groups were found [44]. Would they have found a difference if they had screened for sub-groups? Would a screening instrument as the one described in Haldorsen's study [42] or could perhaps the use of ARM and the information this study provides give guidance to who might benefit better from what?

It might be useful to further investigate who would take responsibility and benefit from, for example, community based musculoskeletal health interventions or self-care programs provided by a physiotherapist. Might it be that those with an external attitude towards responsibility for musculoskeletal disorders would be more likely to benefit from a structured and controlled intervention?

Research on beliefs about responsibility is needed, as there is little information on the benefits of different approaches in preventive care and treatment for musculoskeletal disorders. Beliefs about responsibility could possibly influence clinical practice, policy and funding in both treatment and research, which has been discussed previously in the area of substance use disorders [45].

Future research should explore attitudes towards the responsibility for musculoskeletal disorders of health care providers, where Toombs (1987) described physicians and patients encountering the experience of illness from different "worlds" [46]. Parental high concerns about illness and inadequate beliefs in antibiotics led to more physician consultations and prescriptions for children who had respiratory tract infections [47]. Negative illness attitudes were also independently associated to more consultations in primary care over a 5-year period [48]. A mismatch between professional and patient beliefs may be a partial explanation for the generally poor management of chronic musculoskeletal pain [4]. Can intervention studies with an active approach towards agreement between the provider and patient as to the responsibility for musculoskeletal disorders affect rehabilitation outcome or reduce recurrences? Could an active cognitive approach towards a more internal attitude have such an effect? Future research should also address the need for a deeper understanding of how attitudes towards responsibility for musculoskeletal disorders are formed. How do people reason their allocation of responsibility for management of musculoskeletal disorders?

## Conclusion

In conclusion, a majority of the studied population showed attitudes towards responsibility for the management of musculoskeletal disorders that indicate that these disorders are a matter to be addressed by the individual and also to some extent a responsibility that should be shared by medical professionals.

Associated background variables were mainly physical inactivity, musculoskeletal disorder related sick leave and no education beyond the compulsory level, which increased the odds of attributing responsibility externally, i.e. placing responsibility on someone or something else. These variables may be of interest and should be considered when planning the prevention, treatment and management of musculoskeletal disorders. Attitudes have implications for behaviour. If people believe themselves to be active in the management of musculoskeletal disorders, they may be more responsive to suggestions about self-care, which in turn might have implications for the society's health care costs.

## Competing interests

The authors declare that they have no competing interests.

## Authors' contributions

MEHL involved in conception and design, obtaining grants, development of questionnaire, acquisition, preparation of dataset, statistical analyses and interpretation of data, drafting the article. LAN involved in conception and design, development of questionnaire, statistical analyses and interpretation of data, substantial contribution in revising the article for important intellectual content. Both authors have read and approved the final manuscript.

## Pre-publication history

The pre-publication history for this paper can be accessed here:



## Supplementary Material

Additional file 1 Appendix. Items of "Attitudes regarding Responsibility for Musculoskeletal disorders" (ARM) clustered by subscales (English translation). Click here for file
