# Supplementary material for: Responsibility for managing musculoskeletal disorders – A cross-sectional postal survey of attitudes
Source: BMC Musculoskelet Disord. 2008 Aug 5;9:110. doi: 10.1186/1471-2474-9-110 (PMC2533659; doi:10.1186/1471-2474-9-110)
Supplement: Additional file 1 — Appendix. Items of "Attitudes regarding Responsibility for Musculoskeletal disorders" (ARM) clustered by subscales (English translation). [file 1471-2474-9-110-S1.doc]

Items of “Attitudes regarding Responsibility for Musculoskeletal disorders”^1^ (ARM) clustered by subscales (English translation).

“Responsibility Self-Active”

3. By training my muscles and learning to use my body correctly, I could alleviate discomforts in my muscles and joints.

5. By learning a particular technique, I could, for example, reduce my back discomfort myself.

6. If, for example, I have experienced pain in my knee, I take preventive action to avoid getting this pain again.

7. If I experience pain somewhere, it is of course my responsibility to ensure that I get well.

8. I make time for exercise to reduce the risk of muscle and joint problems.

12. I am aware of the fact that my body has to hold up for many more years, and I do my best to take care of it.

“Responsibility Out of my hands”

1. There is nothing I can do to relieve the pain/discomfort in my muscles and joints.

4. I don’t know any way to prevent discomfort in my muscles and joints.

13. I do not think that whatever I do has any effect on, for example, shoulder pain.

“Responsibility Employer”

2. If, for example, I experience pain in my shoulders, it is because my employer has not taken necessary measures to make my work easier.

11. Only the employer can take the appropriate preventive measures to ensure that muscle and joint problems do not occur in the work place.

15. The only way to avoid disorders of the muscles and joints is to have more people/staff sharing physically heavy work.

“Responsibility (Medical) Professionals”

9. If I experience back pain, I seek the advice of a physician or some other person until I find someone who can cure me.

10. If, for example, I have knee problems, I turn to someone who can take corrective measures.

14. If, for example, I experience back pain, I need professional help to get medication or some other form of treatment.

^1^Larsson MEH, Nordholm LA: **Attitudes regarding responsibility for musculoskeletal disorders - Instrument development**. *Physiotherapy Theory and Practice* 2004, **20**:187-199.
